# Correction to Targeted Precision Cryotherapy for Acne Vulgaris

**DOI:** 10.1111/srt.70313

**Published:** 2025-12-21

J. Y. Hong, K. R. Kim, H. J. Kim, J. Seok, and K. Y. Park, “Targeted Precision Cryotherapy for Acne Vulgaris,” *Skin Research and Technology* 30, no. 9 (2024): e70045, https://doi.org/10.1111/srt.70045.

The published article contains incomplete or inaccurate statements. The following sections have been added to supplement the original content or replace inaccurate published statements.

The legend for Figure 1 should have read: “Non‐contact type cooling device, TargetCool; image officially provided by *RecensMedical Inc*. for academic use.”

The legend for Figure 3 should have read: “Representative case demonstrating noticeable improvement following cryotherapy. (A) Baseline (Visit 1): IGA 3, EI 3. (B) After treatment (Visit 4): IGA 1, EI 1, global evaluation scale 1, and participant satisfaction score 7. Visits occurred at 1‐week intervals.”

In section 2.1, “Ethics approval”, the text should have read: “All patients provided informed consent for participation in the study and for publication of the results”.

In “CONFLICT OF INTEREST STATEMENT”, the text should have read: “This study was funded by *RecensMedical Inc*.”

The authors apologize for these oversights and for the inconvenience they may have caused.

